# Recurrent Symptomatic Renal Angiomyolipoma After Selective Arterial Embolization: A Case Report

**DOI:** 10.7759/cureus.102222

**Published:** 2026-01-24

**Authors:** Anas E Ahmed, Abdulelah M Almuaythir, Nawaf M Alqahtani, Abdullah A AlAzzaz, Meshal A Alqahtani

**Affiliations:** 1 Community Medicine, Jazan University, Jazan, SAU; 2 College of Medicine, Prince Sattam bin Abdulaziz University, Al Kharj, SAU; 3 College of Medicine, King Saud University, Riyadh, SAU; 4 College of Medicine, King Khalid University, Abha, SAU

**Keywords:** computed tomography, digital subtraction angiography, nephron-sparing treatment, recurrent abdominal pain, renal angiomyolipoma, renal tumor, residual tumor vascularity, selective arterial embolization

## Abstract

A middle-aged woman with a history of a large right renal angiomyolipoma (AML), previously treated with selective arterial embolization, presented to our facility with a sudden onset of recurrent right-sided abdominal pain. Despite a prolonged asymptomatic period following her initial procedure, clinical evaluation and laboratory testing were initiated to investigate the recurrence. Cross-sectional imaging revealed an increase in the size of the residual mass and areas of hyperdensity, suggesting minor recurrent hemorrhage. Subsequent digital subtraction angiography was performed, which provided a definitive characterization of the vascular supply, demonstrating partial recanalization of previously embolized vessels and a prominent residual tumor blush.

Following a multidisciplinary review, the patient underwent a repeat selective arterial embolization. The procedure was successful, achieving total devascularization of the target area. Post procedure, the patient experienced complete symptom resolution. Long-term follow-up confirmed sustained clinical stability and radiological improvement, demonstrating that repeat embolization is an effective nephron-sparing strategy for managing late-stage recanalization in renal AML.

## Introduction

Renal angiomyolipoma (AML) is a benign mesenchymal tumor composed of varying proportions of dysmorphic blood vessels, smooth muscle, and adipose tissue [1-3]. It represents the most common benign renal neoplasm and occurs either sporadically or in association with tuberous sclerosis complex [2,3]. Sporadic AMLs are typically unilateral, solitary, and more common in middle-aged women [1-4]. Although many lesions remain asymptomatic and are discovered incidentally on imaging, larger tumors carry a risk of spontaneous hemorrhage due to their abnormal vascular architecture, which may result in life-threatening retroperitoneal bleeding [3,4]. The risk of hemorrhage increases with tumor size, presence of aneurysmal vessels, and hormonal influences [4,5].

Selective arterial embolization has become an established minimally invasive treatment option for symptomatic or large renal AMLs, offering effective hemorrhage control while preserving renal parenchyma [1-5]. It is commonly used as a first-line therapy in acute bleeding and as a prophylactic measure in high-risk lesions [3,4]. Despite high technical and clinical success rates, residual or recurrent tumor vascularity and symptom recurrence have been reported, necessitating long-term surveillance [3,6]. Recurrent abdominal or flank pain in patients previously treated with embolization poses a diagnostic and management challenge, requiring careful clinical and radiological evaluation [1-7].

This case highlights the importance of recognizing symptomatic residual renal AML following embolization and underscores the role of repeat imaging and angiographic assessment in guiding appropriate management.

## Case presentation

A 45-year-old woman with no significant comorbidities presented to the Emergency Department with recurrent right-sided abdominal pain of three days' duration. The pain was described as dull, constant, progressively worsening, localized to the right flank and right upper quadrant, and occasionally radiating to the back. It was associated with nausea but no vomiting, fever, urinary symptoms, hematuria, weight loss, or bowel habit changes. There was no history of recent trauma.

Her past medical history was notable for a right renal AML diagnosed three years earlier, which had been treated with selective arterial embolization following an episode of acute flank pain and radiological evidence of intratumoral hemorrhage. After embolization, she had remained clinically well and was considered medically free, with regular outpatient follow-up showing stable disease. She was not known to have tuberous sclerosis complex, and there was no family history of renal tumors or genetic disorders. She was not taking any regular medications and had no known drug allergies.

On presentation, the patient was alert and oriented, in mild distress due to pain. Her vital signs were stable, with a blood pressure of 122/76 millimeters of mercury, pulse rate of 88 beats per minute, respiratory rate of 18 breaths per minute, temperature of 36.8 degrees Celsius, and oxygen saturation of 99% on room air. Abdominal examination revealed localized tenderness over the right flank and right upper quadrant without guarding or rebound tenderness. No palpable abdominal mass was appreciated. Bowel sounds were normal. There was no costovertebral angle tenderness on the left and only mild tenderness on the right. Cardiovascular, respiratory, and neurological examinations were unremarkable. There were no cutaneous stigmata suggestive of tuberous sclerosis.

Initial laboratory investigations demonstrated a hemoglobin level of 12.6 grams per deciliter, white blood cell count of 7.9 × 10⁹ per liter, and platelet count of 265 × 10⁹ per liter. Renal function tests were within normal limits, with a serum creatinine of 0.82 milligrams per deciliter and blood urea nitrogen of 14 milligrams per deciliter. Liver function tests and serum electrolytes were normal. C-reactive protein was mildly elevated at 8 milligrams per liter. Urinalysis showed no hematuria, proteinuria, or signs of infection. A pregnancy test was negative.

**Figure 1:**
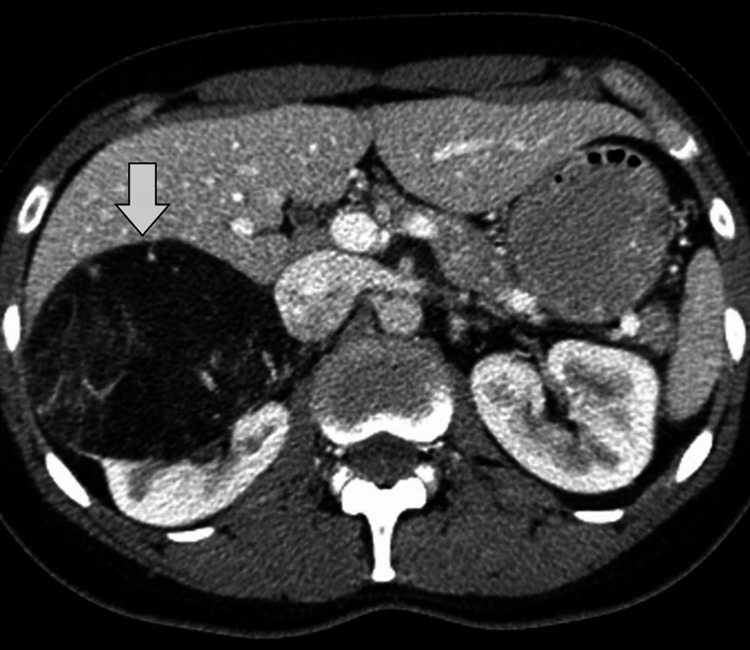
Renal angiomyolipoma on computed tomography Axial contrast-enhanced computed tomography (CT) image of the abdomen demonstrates a well-defined fat-containing lesion (arrow) arising from the right kidney, characterized by areas of negative attenuation consistent with renal angiomyolipoma.

Given her history and current symptoms, a contrast-enhanced computed tomography (CT) scan of the abdomen and pelvis was performed. Review of prior imaging before embolization showed a well-defined, predominantly fat-containing lesion arising from the lower pole of the right kidney, measuring approximately 7.5 cm in maximum diameter, with areas of high attenuation consistent with acute hemorrhage. There was mild perinephric stranding but no large retroperitoneal hematoma. No hydronephrosis or renal vein involvement was observed (Figure 1).

**Figure 2:**
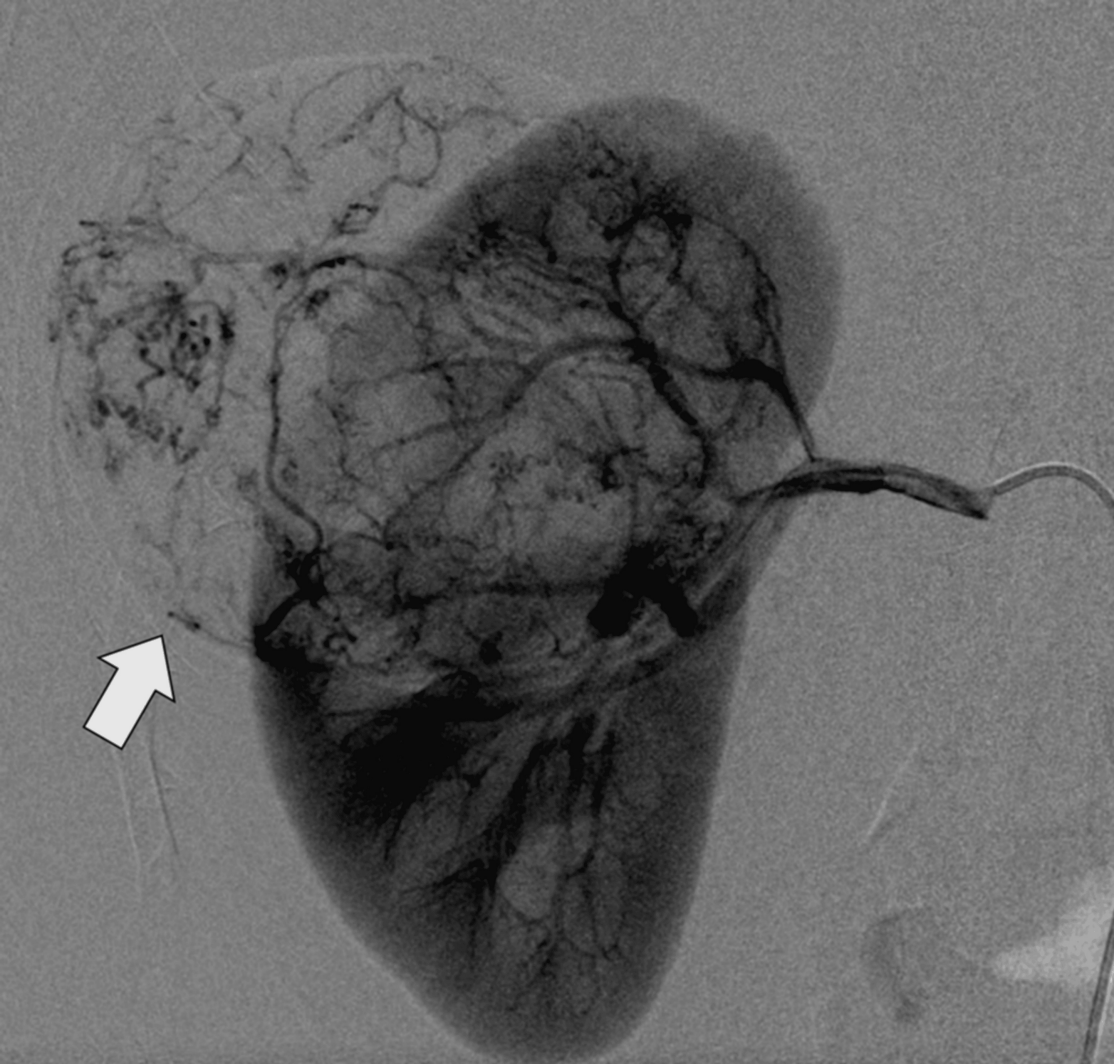
Angiographic appearance of renal angiomyolipoma Digital subtraction angiography (DSA) image shows a highly vascular lesion (arrow) in the right kidney with prominent tumor blush, corresponding to the angiomyolipoma prior to embolization.

To further evaluate the vascular status of the lesion and exclude active bleeding, digital subtraction angiography (DSA) was performed. This demonstrated a previously embolized right renal arterial branch supplying the AML, with evidence of partial recanalization and small residual tumor blush, but no frank contrast extravasation at the time of imaging (Figure 2).

The patient was managed initially with conservative measures, including analgesia, intravenous fluids, and close monitoring of vital signs and hemoglobin levels. Given her recurrent symptoms and angiographic evidence of residual vascularity, a multidisciplinary team decision was made to proceed with repeat selective arterial embolization. The procedure was performed successfully without complications, achieving complete devascularization of the residual lesion. During her hospital course, the patient's pain gradually improved, and serial hemoglobin measurements remained stable. Renal function was preserved throughout admission. She was discharged home on the fourth hospital day with oral analgesics and clear instructions for follow-up.

**Figure 3:**
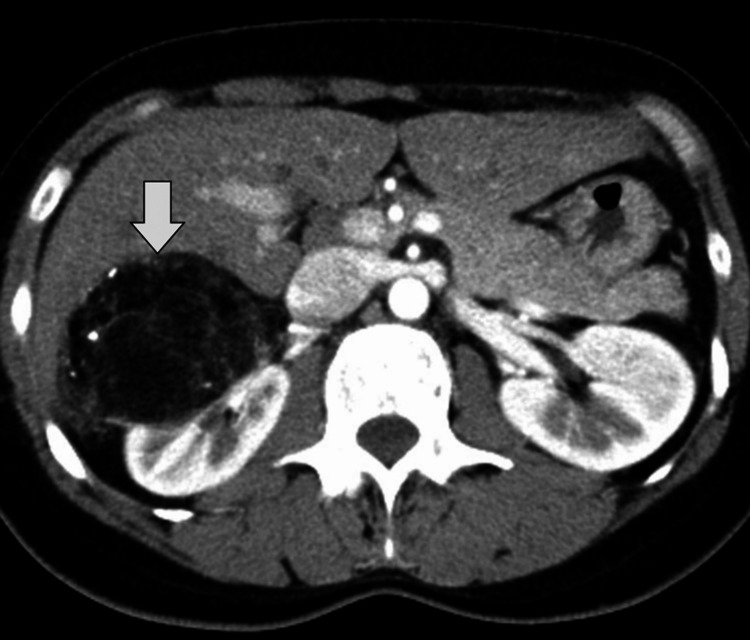
Post-embolization follow-up imaging Axial contrast-enhanced CT image obtained after embolization demonstrates a marked interval reduction in the size of the right renal angiomyolipoma (arrow), indicating successful treatment response.

At follow-up visits at one month and six months post-procedure, the patient remained asymptomatic. Repeat contrast-enhanced CT imaging demonstrated further reduction in the size of the AML with no evidence of residual enhancement or hemorrhage (Figure 3). Renal function tests remained normal, and the patient returned to her baseline level of activity without recurrence of abdominal pain.

## Discussion

Renal AML is a benign renal neoplasm with a well-recognized potential for clinically significant morbidity despite its nonmalignant nature [1-7]. The present case underscores several important aspects of AML behavior, management, and follow-up that are highly relevant to contemporary clinical practice. Although selective arterial embolization is widely accepted as an effective and nephron-sparing treatment for symptomatic or high-risk AMLs [5-9], this case highlights that symptom recurrence may occur even years after an initially successful intervention, emphasizing the need for long-term vigilance.

One of the most clinically relevant issues in AML management is hemorrhagic risk [4,5]. The abnormal vascular component of AMLs, characterized by tortuous, aneurysm-prone vessels lacking normal elastic tissue, predisposes these tumors to spontaneous bleeding [3,6]. While tumor size greater than 4 cm has historically been used as a threshold for intervention, more recent evidence suggests that vascular features such as intratumoral aneurysm size and tumor growth rate may be more accurate predictors of bleeding risk [4-7]. In the present patient, initial embolization successfully controlled hemorrhage and reduced tumor vascularity; however, partial recanalization and residual vascular supply were later identified on angiography.

Recurrent abdominal or flank pain following embolization presents a diagnostic challenge [5-8]. Pain may result from post-embolization syndrome, tumor necrosis, ischemic changes in adjacent renal tissue, or recurrent microhemorrhage within residual AML tissue [4-9]. In this case, the absence of systemic inflammatory signs, stable hemoglobin levels, and subtle imaging findings favored minor recurrent intralesional bleeding rather than acute massive hemorrhage or infection [7,8]. The role of contrast-enhanced CT was pivotal in identifying subtle changes suggestive of recurrence, while DSA provided definitive assessment of residual tumor vascularity.

Differentiating residual or recurrent AML from malignant renal neoplasms remains an important consideration, particularly in cases with atypical imaging features or persistent symptoms [4-7]. Although the presence of macroscopic fat strongly supports the diagnosis of AML, fat-poor or epithelioid variants can mimic renal cell carcinoma and carry malignant potential [1,9]. In the current case, the consistent imaging appearance over time and angiographic findings supported a benign etiology, obviating the need for surgical biopsy or nephrectomy.

From a therapeutic standpoint, repeat selective arterial embolization was an appropriate and effective management strategy [2-8]. Compared with surgical options, embolization offers renal parenchymal preservation, reduced morbidity, and shorter recovery time [1,4]. Repeat embolization is safe and effective in cases of residual or recurrent AML vascularity, particularly in patients without tuberous sclerosis complex and with preserved renal function.

Long-term follow-up is a critical but sometimes underemphasized component of AML management [1-5]. There is no universal consensus regarding optimal surveillance intervals following embolization; however, periodic imaging is generally recommended, especially in patients with large tumors, incomplete devascularization, or recurrent symptoms [4-9]. This case supports an individualized follow-up strategy based on patient-specific risk factors and clinical course.

## Conclusions

This case highlights that renal AML, despite its benign pathology, can demonstrate recurrent symptomatic behavior even after initially successful selective arterial embolization. Recurrent abdominal or flank pain in such patients should prompt thorough clinical and radiological reassessment, as residual or recanalized tumor vascularity may lead to ongoing microhemorrhage or symptom recurrence. Cross-sectional imaging complemented by angiographic evaluation plays a crucial role in accurate diagnosis and therapeutic planning. Repeat embolization represents a safe, effective, and nephron-sparing treatment option capable of achieving durable symptom control while preserving renal function. Long-term, individualized surveillance and a multidisciplinary approach are essential to identify and manage late complications or recurrence promptly.
